# Hepatitis A outbreak in HIV-infected MSM and in PrEP-using MSM despite a high level of immunity, Lyon, France, January to June 2017

**DOI:** 10.2807/1560-7917.ES.2017.22.48.17-00742

**Published:** 2017-11-30

**Authors:** Caroline Charre, Christophe Ramière, Anne-Marie Roque-Afonso, Christian Chidiac, Fabien Zoulim, Matthieu Godinot, Joseph Koffi, Caroline Scholtès, Jean-Michel Livrozet, HAV Lyon Study Group, Laurent Cotte

**Affiliations:** 1 Laboratoire de Virologie, Hôpital de la Croix-Rousse, Hospices Civils de Lyon, Lyon, France; 2 Université de Lyon, Lyon, France; 3 CIRI, International Center for Infectiology Research, Université de Lyon, Lyon, France; 4 National reference centre for hepatitis A virus (Centre national de référence du virus de l’hépatite A), Virologie, Hôpital Paul Brousse, AP-HP, Villejuif, France; 5 Service de maladie infectieuse et tropicale, Hôpital de la Croix Rousse, Hospices Civils de Lyon, Lyon, France; 6 Service d’Hépatologie et de gastroentérologie, Hôpital de la Croix-Rousse, Hospices Civils de Lyon, Lyon, France; 7 INSERM, U871, Lyon, France; 8 Service de maladie infectieuse et tropicale, Hôpital Edouard Herriot, Hospices Civils de Lyon, Lyon, France; 9 The members of the HAV Lyon Study Group are listed at the end of the article

**Keywords:** Hepatitis A, hepatitis A virus, human immunodeficiency virus - HIV, men who have sex with men - MSM, preexposure prophylaxis - PrEP, outbreaks, sexually transmitted disease - STD

## Abstract

Since 2016, an increase in the number of hepatitis A cases affecting mainly men who have sex with men (MSM) has been reported in low endemic countries in Europe. We calculated the attack rate in Lyon, France, in populations considered at high-risk: HIV-infected MSM and HIV-negative MSM receiving HIV pre-exposure prophylaxis (PrEP). In these populations, high level of immunity did not prevent the outbreak, indicating that vaccination should be reinforced, particularly in younger individuals.

Several outbreaks of acute hepatitis A among men who have sex with men (MSM) have been recently reported in different European countries [1-4]. Since the end of 2016, an important increase in the number of acute hepatitis A cases in MSM has been also notified in France through the national mandatory reporting system [5]. The aim of this study was to evaluate the proportion of hepatitis A virus (HAV)-susceptible individuals and the attack rate of acute hepatitis A in HIV-infected MSM and in HIV-negative MSM receiving HIV pre-exposure prophylaxis (PrEP).

## Case definition and cohort description

The infectious diseases department of the Hospices Civils de Lyon follows ca 3,800 HIV-infected patients per year, representing 94% of HIV-infected patients followed in the Rhône department. Additionally, 415 MSM who used PrEP in the Rhône department were followed in the infectious diseases department during the study period. All cases of acute hepatitis A diagnosed in the Hospices Civils de Lyon virology laboratory between 1 January and 30 June 2017 were included. Diagnosis was based on the detection of serum HAV-specific IgM antibodies (ADVIA Centaur HAV assays, Siemens, Canada) along with elevated liver enzymes. HAV sequencing from IgM-positive samples was performed by the HAV national reference centre in Villejuif, France, as previously reported [6].

All 2,023 HIV-infected MSM and 415 PrEP users followed during the period were enrolled. Demographics (age, HIV status, PrEP use), HAV and hepatitis B virus (HBV) serological status, previous history of HAV infection and HAV vaccination history were retrieved from the clinical database and are compiled in the Table. The proportion of HAV-susceptible patients was determined based on medical, serological and vaccination history. Criteria for HAV immunity were: (i) past documented acute hepatitis A or (ii) past positive test for HAV total antibodies or (iii) administration of at least one dose of hepatitis A vaccine before January 2017. Evaluation of the attack rate in HIV-infected susceptible patients was determined by category of age. To take into account the patients with unknown HAV immune status, sensitivity analyses were performed assuming that patients with unknown HAV immune status were considered as susceptible (best case scenario), and that patients with unknown HAV immune status were considered as immune (worst case scenario). The hepatitis A attack rate was not determined by category of age in PrEP users due to the limited number of cases. HAV cases in HIV-negative MSM not enrolled in the PrEP programme were not considered for the determination of the attack rate, since the denominator for this population is unknown. The study was approved by the local ethics committee.

**Table:** Characteristics of HIV-infected MSM (n = 2,023) and MSM PrEP users (n = 415), January–June 2017, Lyon, France

Characteristic	HIV-infected MSM (n = 2,023)		MSM PrEP users (n = 415)	
**Age** **median (IQR)**	**49** **(40–57)**		**36** **(29–44)**	
**CD4 cells count / mm^3^** **median (IQR)**	**676** **(515–857)**		**NA**	
**Antiretroviral treatment**	**n**	**%**	**NA**	
	2,006/2,022^a^	99.2		
**HIV viral load < 40 copies/mL**	**n**	**%**		
	815/1,963^a^	92.5		
**HBV status**	**n**	**%**	**n**	**%**
Chronic hepatitis B	67	3.3	1	0.2
Cured hepatitis	693	34.3	18	4.3
Vaccinated	1,041	51.5	349	84.1
Non immune	175	8.6	31	7.5
Unknown	47	2.3	16	3.9
**HAV status**	**n**	**%**	**n**	**%**
Immune	1,219	60.3	305	73.5
• Vaccinated	417	20.6	158	38.1
• Previous history of hepatitis A	109	5.4	14	3.4
• Positive serology	1,037	51.3	210	50.6
Non immune	442	21.8	101	24.3
Unknown status	362	17.9	9	2.2
**Acute hepatitis A during outbreak (attack rate %)**				
Best scenario	2		2.7	
Worst scenario	3.8		3	
**Hepatitis A strain (n = 19)**				
1a_RIVM_HAV16–90 (EuroPride)	6		0	
1a_VRD_521_2016 (UK/Spain)	10		2	
1a_V16–25801	0		1	

IQR: interquartile range; MSM: men who have sex with men; NA: not applicable; PrEP: pre-exposure prophylaxis; UK: United Kingdom.

^a^ Number of patients with information available for this variable.

## Description of the outbreak

**Figure 1:**
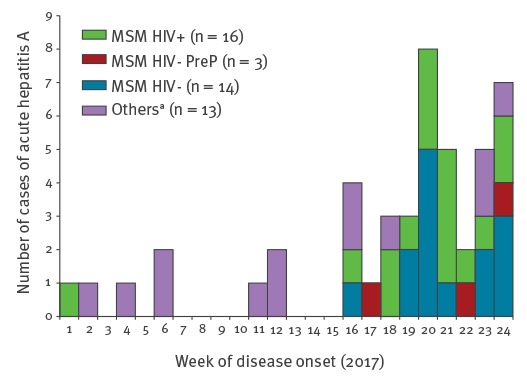
Epidemic curve of total hepatitis A cases, January–June 2017, Lyon, France (n = 46)

From 1 January 2017 to 30 June 2017, 46 cases of acute hepatitis A were diagnosed in the laboratory (Figure 1) among whom 34 occurred between May and June. Two cases occurred in children under 15 years old. Among 44 adult cases, 38 were men (sex ratio M/F: 6.3); 33 were MSM, including 17 HIV-negative (among whom three were PrEP users) and 16 HIV-infected. Two cases in HIV-infected MSM who did not live in the Rhône department were excluded from the attack rate analysis. In all but one case occurring in MSM, sequencing identified one of the three epidemic strains circulating among MSM in many European countries [2-4]: 1a_VRD_521_2016 (UK/Spain; 18/33), 1a_RIVM_HAV16–090 (EuroPride; 12/33), and 1a_V16–25801 (2/33) (Table).

HIV +: HIV infected; HIV-: HIV negative; MSM: men who have sex with men; PrEP: pre-exposure prophylaxis

^a ^The category ‘Others’ includes women, children and heterosexual men.

**Figure 2:**
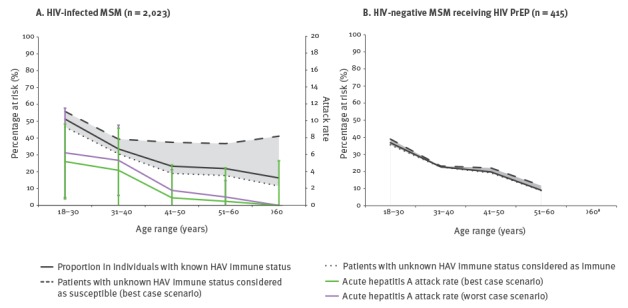
Proportion of individuals susceptible to HAV and acute hepatitis A attack rate according to age, January–June 2017, Lyon, France

HAV: hepatitis A virus; MSM: men who have sex with men; PrEP: pre-exposure prophylaxis.

^a^ Number too small to calculate the percentage at risk.

HIV-infected MSM were significantly older than PrEP users (p < 0.001). Among those with information available, the proportion of HAV-susceptible patients was not significantly different between groups (HIV-infected MSM: 26.6%, PrEP users: 24.9%, p = 0.48). The attack rate irrespective of age was similar in HIV-infected MSM (best case scenario: 2%; worst case scenario: 3.8%) and in PrEP users (best case scenario: 2.7%; worst case scenario: 3%; Table). HAV susceptibility in patients with a known immune status was higher in patients aged 18–30 years, both among PrEP users (36%) and in HIV-infected MSM (47%) and decreased with age. The best and worst case scenario gave results that were close, except for the older HIV-infected group, in which a greater number of unknown status led to an increase in susceptibility in the best case scenario (Figure 2). The attack rate in HIV-infected MSM was highest in those aged 18–30 years (best case scenario: 5.2%; worst case scenario: 6.3%) and decreased with age to reach 0% in those aged 60 years or more.

Black lines: proportion of individuals susceptible to HAV.

Error bars: 95% confidence intervals.

## Discussion

In high-income countries the prevalence of anti-HAV antibodies in the general population is usually low (< 50% by the age of 30 years) [7] Therefore, the high proportion of susceptible individuals among adults could theoretically allow transmission, but usually hygiene measures limit the circulation of the virus and the risk of acquiring infection remains low. However, if HAV is introduced in groups at particular high-risk of transmission, outbreaks may occur according to level of immunity. Due to faeco-oral transmission during sexual activities, in particular bucco-anal, digital-anal, digital-rectal, and genito-oral activities following anal sex, MSM are at a high risk of HAV transmission. Moreover, as HAV transmission from sharing needles has also been described [8], intravenous injection of recreational drugs along with anal intercourse (also known as slamming) may increase the risk of HAV transmission in some groups of MSM. For these reasons, anti-HAV vaccination is routinely recommended in France in HIV-infected MSM and PrEP users [9], contributing to a high level of immunity compared with the general population. For example, in a recent survey, 78% of adults aged 20–29 years were susceptible to HAV, as opposed to 31% of HIV-infected MSM and 29% of PrEP users in the present study [10]. Nevertheless, this high level of immunity did not prevent HAV outbreak in the present study.

Between 1 January 2017 and 30 June 2017, 57 cases of acute hepatitis A were notified through the mandatory reporting system in the Rhône department. Among these 57 cases, 46 were diagnosed in our laboratory, suggesting a satisfying representativeness of the cases analysed here for the overall outbreak in the department. As previously reported, the outbreak described here affected mainly MSM with a similar attack rate in HIV-positive MSM and PrEP users, suggesting a comparable transmissibility in both groups considered at high risk of transmission.

One limitation of the study is that HAV immune status remained unknown in 17.9% of HIV-infected MSM and in 2.2% of PrEP users. However, in the best case scenario sensitivity analysis attack rates were high, confirming that both populations were engaged in at-risk sexual behaviour, as previously described [11,12]. Another limitation is that HAV immune status was not systematically confirmed serologically. Moreover, a single dose of hepatitis A vaccine may not be sufficient to provide immunity in a HIV-positive patients [13]. However, during the study period, no case of acute hepatitis A was reported in our centre among patients who received a single dose of vaccine.

Predictions from a modelling study suggested that population immunity must exceed 70% to prevent future person-to-person transmission of hepatitis A virus among MSM [14]. According to this model, the immunity level in the study population should have conferred protection against HAV outbreaks. However, HAV-susceptibility was much higher in younger individuals, which is likely to explain the higher attack rate observed before 40 years of age. Additionally, differences in risk practices among age groups may also have influenced transmission of the virus and the attack rate.

As immunity threshold of 70% seems to be insufficient to prevent HAV outbreaks, it should be adjusted considering behavioural characteristics, socio-demographic characteristics, and different age structures. Promotion of HAV vaccination should be reinforced, especially in young MSM who are at high risk of HAV transmission.

## Notes

**Members of the HAV Lyon Study Group:** **Hôpital de la Croix Rousse, Lyon**: C. Chidiac, L. Cotte, J. Koffi, P. Miailhes, T. Ferry, F. Zoulim, F. Bailly, S. Radenne, M. Maynard-muet, P. Lack, K. Vandevoorde, G. Ouziel, M. Levrero, D. Poinsot, M. O'brien, A. Louf-durier, V. Escuret, I. Schuffenecker, M.A. Trabaud, C. Scholtès, C. Ramière, C. Achard, C. Charre.
**Hôpital Edouard Herriot, Lyon**: J.M. Livrozet, M. Godinot, D. Makhloufi, J. Dumortier, C.A. Durel, A. Hot, Y. Khennouf.
